# Nanodetection of Head and Neck Cancer on Titanium Oxide Sensing Surface

**DOI:** 10.1186/s11671-020-3262-x

**Published:** 2020-02-03

**Authors:** Yu Wang, Yan Guo, Jianguang Lu, Yanan Sun, Xiaoguang Yu, Subash C. B. Gopinath, Thangavel Lakshmipriya, Yuan Seng Wu, Chao Wang

**Affiliations:** 1 Department of Otolaryngology-Head and Neck Surgery, The Second Affiliated Hospital of Harbin Medical University, Harbin, 150081 Heilongjiang China; 2 Department of Biochemistry and Molecular Biology, Harbin Medical University, Harbin, 150081 Heilongjiang China; 3 School of Bioprocess Engineering, Universiti Malaysia Perlis, 02600 Arau, Perlis Malaysia; 4 Institute of Nano Electronic Engineering, Universiti Malaysia Perlis, 01000 Kangar, Perlis Malaysia; 5 Department of Biochemistry, Faculty of Medicine and Biomedical Sciences, MAHSA University, Bandar Saujana Putra, 42610 Jenjarom, Selangor Malaysia

**Keywords:** Squamous cell carcinoma antigen, Interdigitated electrode, Titanium oxide, Gold star, Circulating biomarker

## Abstract

Head and neck cancer is a heterogeneous disease, originating in the squamous cells lining the larynx (voice box), mouth, pharynx (throat), nasal cavity and salivary glands. Head and neck cancer diagnosis at the later stage is greatly influencing the survival rate of the patient. It makes a mandatory situation to identify this cancer at the earlier stages of development with a suitable biomarker. Squamous cell carcinoma antigen (SCC-Ag) is a circulating serum tumour biomarker, and the elevated level has been found in the head and neck cancer patients and highly correlated with the tumour volume. The present research was carried out to detect and quantify the level of SCC-Ag on titanium oxide (TiO_2_)-modified interdigitated electrode sensor (IDE) by SCC-Ag antibody. The detection of SCC-Ag was found at the level of 100 fM, while it was improved to 10 fM when the antibody was conjugated with gold nanostar, representing a 10-fold improvement. Interestingly, this enhancement in sensitivity is 1000-folds higher than other substrates. Moreover, the specificity analysis was carried out using two different control proteins and noticed that the antibody only recognised SCC-Ag, indicating the specific detection on IDE-TiO_2_ sensing surface.

## Introduction

Head and neck cancer shows the abnormal cell growth in the area of the head and neck and widely reported. It originates from the throat, mouth, mucosa, epithelia of the oral cavity, salivary glands and nasal cavity [1]; is the sixth most commonly reported cancer worldwide; and affects more than 644,000 people every year [2]. Most of the affected patients are diagnosed at the advanced stages and highly affect their survival. Early-stage identification of head and neck cancer is mandatory to improve the survival and lifestyle. Serologic tumour markers have been used to diagnose and manage the follow-up treatment of head and neck cancer. The squamous cell releases a predominant squamous cell carcinoma antigen (SCC-Ag), its presence elevated in cancer patients and SCC-Ag has shown to be a promising tumour marker with squamous cell-related cancers such as gynaecologic, lung, oesophageal and anal cancers [3, 4]. Considering head and neck cancer, higher levels of SCC-Ag have been associated with disease metastasis, recurrence and mortality as attested in different studies with cancer patients [5–7]. Researchers have found that serum SCC-Ag was at a significant risk level for the cancers in the hypopharynx, oral cavity and larynx [8, 9]. In addition, there was a correlation between SCC-Ag level and the tumour volume in head and neck cancer patients [10]. It is wise to quantify the level of SCC-Ag to identify the condition of head and neck cancer, in order to provide the earlier treatment. The current research was focused on detecting SCC-Ag at its lower level using the nanoparticle on interdigitated electrode (IDE) sensor by SCC-Ag antibody.

IDE is an electrochemical biosensor having promising features such as low-cost, portable and sensitive, makes a wide range of applications, in particular with environmental monitoring and medical diagnosis [11, 12]. Enhancing the electrical property on the sensing surface improves the detection of biomolecules. Nanomaterial application has been broadly used in the biosensor to enhance the biomolecular detection on sensing surfaces. Nanomaterials are smaller in size, have larger surface area, have good thermal and electrical conductivity, are compatible with biomolecules, and show a tremendous capability to be applied in the field of biosensor [13, 14]. Nanomaterial has been applied in two different ways for purposes: one is surface functionalization and another is conjugating the analyte or target in order to improve the detection [15]. Gold is one of the well-established nanomaterials and applied in various sensors, which include surface plasmon resonance, waveguide-mode sensor, electrochemical sensor and colorimetry [16–18]. Apart from that, silver, graphene, copper and titanium nanomaterials were also applied in various biomedical applications. As an environment-friendly semiconductor and low cost, titanium oxide (TiO_2_) has a wide bandgap utilized here for the surface modification on IDE to detect SCC-Ag. Owing to the high electrical and optical properties of TiO_2_, it is widely used for super-capacity purpose, photocatalytic and photoelectric conversions [19–23]. Moreover, its nature of hydrophilicity and larger surface area are suitable for the surface modification and help detect the biomolecules at a lower level. In this research, TiO_2_ was coated on IDE sensing surface to enhance the electric flow when the interaction of biomolecules is happening. To improve the detection of SCC-Ag, an antibody was conjugated with gold nanostar (GNS antibody) and immobilized on TiO_2_-coated surface. Since it has been proven that gold nanomaterial-conjugated biomolecules exhibit a higher stability and provide the properly oriented surface-immobilized biomolecules, it has the ability to improve the limit of detection [24, 25]. In addition, more biomolecules can be immobilized on a single gold particle, which leads to attract the elevated levels of the target molecule. In this work, two different nanomaterials, namely TiO_2_ (for surface modification) and GNS (for antibody conjugation), were used to improve the detection of SCC-Ag on IDE sensing surface. The application of GNS is expected to enhance the performance with the current sensor by its larger surface to capture the higher number of antibodies.

## Materials and Methods

### Reagents and Biomolecules

SCC antigen (a glycoprotein with isoforms ranging from 45 to 55 kDa) was purchased from Randox Life Sciences (Malaysia). Anti-SCC antibody was procured from Next Gene (Malaysia). (3-Aminopropyl)triethoxysilane (APTES), ethanolamine, albumin (a major blood protein at 45 mg/mL; 50–70% of blood protein with a molecular weight of 66.5 kDa), phosphate-buffered saline (PBS; pH 7.4) and titanium IV isopropoxide were from Sigma Aldrich (USA). Serpin (a commonly distributed serine protease inhibition with a molecular weight of 40 to 50 kDa) was from Sino Biological (China). Gold nanostar was synthesized as described by Shan et al. [26]. All obtained reagents and chemicals were stored at the recommended by the manufacturer.

### Interdigitated Electrodes Fabrication

The basic design and fabrication of IDE were followed as reported earlier [27]. Initially, the silicon wafer was cleaned by the standard cleaning solutions, and aluminium IDE electrode was deposited by the traditional wet etching method on the silicon wafer. Then, the positive photoresist was deposited on the surface of the silicon wafer, followed by thermal oxidation was done. Deposition of aluminium was performed by the photolithography technique. Three steps were involved, in which step 1 was with 1200 rpm for 10 s then step 2 was with 3500 rpm for 20 s, followed by 500 rpm for 10 s. And then, ultraviolet (UV) light was exposed on the sensing surface to transfer the pattern of IDE onto the sample surface. After that, RD-6 developer was used for 15 s to carry out the developmental process. Photo-resisting was done to eliminate the unexposed regions. The developed sample was baked for 100°°C to clear the unnecessary moisture and improve the adhesion between the SiO_2_ layer and the aluminium. Finally, by using 23 s of aluminium etchant, the unexposed area was removed and cleaned by acetone. The final surface was modified by TiO_2_ to detect SCC-Ag. The fabricated IDE surface was observed under high-power microscopy and 3D nanoprofiler. The images were captured using the associated system at × 50 magnification.

### Coating of TiO_2_ on IDE Sensing Surface

On the fabricated IDE surface, TiO_2_ solution was coated and titanium IV isopropoxide was used as a precursor to prepare the solution of TiO_2_. For that, ethanol was mixed with titanium IV isopropoxide and vigorously stirred for 5 min. And then, the stabilizer (100 μL of acetic acid) was dropped under the stirring condition and then heated on a hot plate at a temperature of 85 °C. The molar ratio mixture was fixed as 9:1:0.1 (ethanol to TIP to acetic acid). After 3 h of mixing, a clear solution was obtained. Upon 24 h of ageing process, the solution was dropped on silicon dioxide (SiO_2_) substrates by using the spin coater at a speed of 2000 rpm. After coating, the surface was dried for 15 min at a temperature of 175 °C and annealed for 1 h at 450 °C. The TiO_2_ thin film gets a sufficient thickness after coating three layers.

### Preparation of GNS-Conjugated Anti-SCC-Ag

SCC-Ag antibody was immobilized on GNS by using the linker 16-mercaptoundecanoic acid (16-MDA). Initially, 5 mM of diluted 16-MDA was mixed with 100 μL of GNS and kept at room temperature (RT) for 30 min. The bound 16-MDA with GNS was removed by centrifugation at 13,000×*g*, 5 min. Then, the collected gold pellet was activated by EDC (400 mM) and NHS (50 mM) with the ratio of 1:1 by incubating for 15 min at room temperature. The unbound EDC and NHS from the solution mix were eliminated by centrifugation at 13,000×*g*, 5 min. The pellet containing the activated GNS was collected to conjugate the antibody. Followed by 200 nM of SCC-Ag antibody was mixed with EDC-NHS-activated GNS and kept at RT for 1 h. Finally, the unbound antibodies were removed by centrifugation at 13,000×*g*, 5 min. The conjugated antibody with GNS was kept at 4 °C for further use, and the conjugation was confirmed by the UV-Vis spectroscopy scanning. Scanning was performed in the region between 480 and 560 nM, and the peak maxima were found.

### Immobilization on GNS Antibody on TiO_2_-IDE Surface

The TiO_2_-coated IDE surface was further modified into amine by APTES to immobilize GNS antibody. APTES with 3% (diluted in 30% ethanol) was dropped on TiO_2_ surface and kept for 3 h at RT. The surface was washed with 30% ethanol three times to remove unbound APTES. To immobilize the antibody, the activation step was followed as mentioned above. The antibody or GNS antibody was dropped on the surfaces and waited for 1 h to complete the immobilization process. Finally, the surface was washed five times by PBS buffer to completely eliminate the unbound antibodies. These antibody or GNS antibody-modified surfaces were used to detect the SCC-Ag and compared. The GNS antibody-immobilized TiO_2_ surface was analysed by atomic force microscopy (AFM), field-emission transmission electron microscopy (FETEM) and energy dispersive X-ray (EDX) analyser as described earlier [15]. AFM observations were at 5 μm scale, whereas SEM was at 100 nM scale operated with 15 kV. The presence of the elements was found by EDX.

### Detection of SCC Antigen on Antibody/Gold Nanostar Antibody Surfaces

To detect SCC-Ag, antibody or gold nanostar antibody-modified TiO_2_-IDE surfaces were blocked by 1 M ethanolamine to mask the antibody-free surface areas and kept for 30 min at RT. On the ethanolamine-blocked surface, 1 nM of SCC-Ag interacted and the current responses were noticed before and after the addition of SCC-Ag. To evaluate the limit of detection, SCC-Ag was titrated from 10 fM to 1 nM and dropped individually on the antibody or GNS antibody-modified surfaces and responses with the current were noted. Experiments were performed in triplicates and calculated the statistics. A linear sweep voltage of 0 to 2 V at 0.01 V step voltage was followed for the measurements. The limit of detection (LOD) was considered the lowest concentration of an analyte (from the calibration line at low concentrations) against the background signal (*S*/*N* = 3:1), in other words, LOD = standard deviation of the baseline + 3*σ*.

### Selective Detection of SCC-Ag

To check the selective interaction of SCC-Ag with its antibody, control experiments were carried out with two different proteins, namely, serpin and albumin. A 1-nM concentration of these control proteins was dropped on antibody or GNS antibody-modified surfaces, and the changes in the current were noticed before and after the interaction. These current levels were compared with the specific detection of SCC-Ag by its antibody and GNS antibody. Other control experimental set-ups include the interaction of SCC-Ag with GNS alone and SCC-Ag with TiO_2_-IDE surface coated by non-immune antibody-labelled GNS. Experiments were performed in triplicates and calculated the statistics. A linear sweep voltage of 0 to 2 V at 0.01 V step voltage was followed for the measurements.

## Results and Discussion

Head and neck cancer has been described as different tumours develop in or around the nose, mouth, larynx and sinuses [28]. Early diagnosis and treatment with a suitable biomarker are mandatory to improve the survival rate of patients. SCC-Ag was found as a suitable serum biomarker for head and neck cancer; herein, the experiments were carried out to detect and quantify the level of SCC-Ag on TiO_2_-modified interdigitated electrode (IDE) sensor by its antibody. TiO_2_ is used here to improve the current response during the interaction of biomolecules. Compared with other nanomaterials, TiO_2_ is considered as attractive in the electrochemical sensor due to its active behaviour on the surface along electrodes and improvement of the electrocatalytic activity. Moreover, it gives more stability to the surface, which yields the repeatability of the response by the electrode and enhancement in the limit of detection by increasing the peak current [29–31]. To utilize this positive feature, in this research, coated TiO_2_ on the IDE surface (IDE-TiO_2_) improves the current flow. Another nanomaterial GNS was used to immobilize anti-SCC-Ag antibody on the IDE-TiO_2_ surface and to enhance the limit of deletion. Since it has been proved that gold-conjugated biomolecule-immobilized surface improves the detection of the target [32, 33], here, SCC-Ag was detected and compared with antibody and GNS antibody modified IDE-TiO_2_ surfaces. As generalized elsewhere, with an increase in the surface area of the nanoparticle, there will be an enhancement in the biomolecular attachment. In this context, GNS has a larger surface compared with the spherical gold nanoparticle. To implement this idea, the current experiment has been performed using GNS to enhance the sensitivity.

### Surface Characterization and GNS Antibody Immobilization

**Fig. 1:**
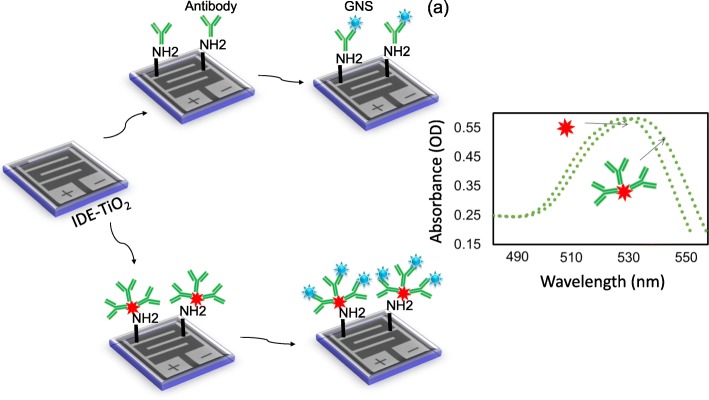
**a** Schematic representation for the detection of SCC-Ag. IDE-TiO_2_ surface was modified into amine by APTES followed by the immobilization of antibody or GNS antibody. The amine group from APTES react carboxyl group on the antibody. SCC-Ag was detected by the interaction at the antigenic region and compared. **b** UV-Vis spectroscopy measurements with GNS. Scanning was in the region between 480 and 560 nM, and the peak maxima were ~ 530 nM. GNS with and without antibody are indicated by the arrows

**Fig. 2:**
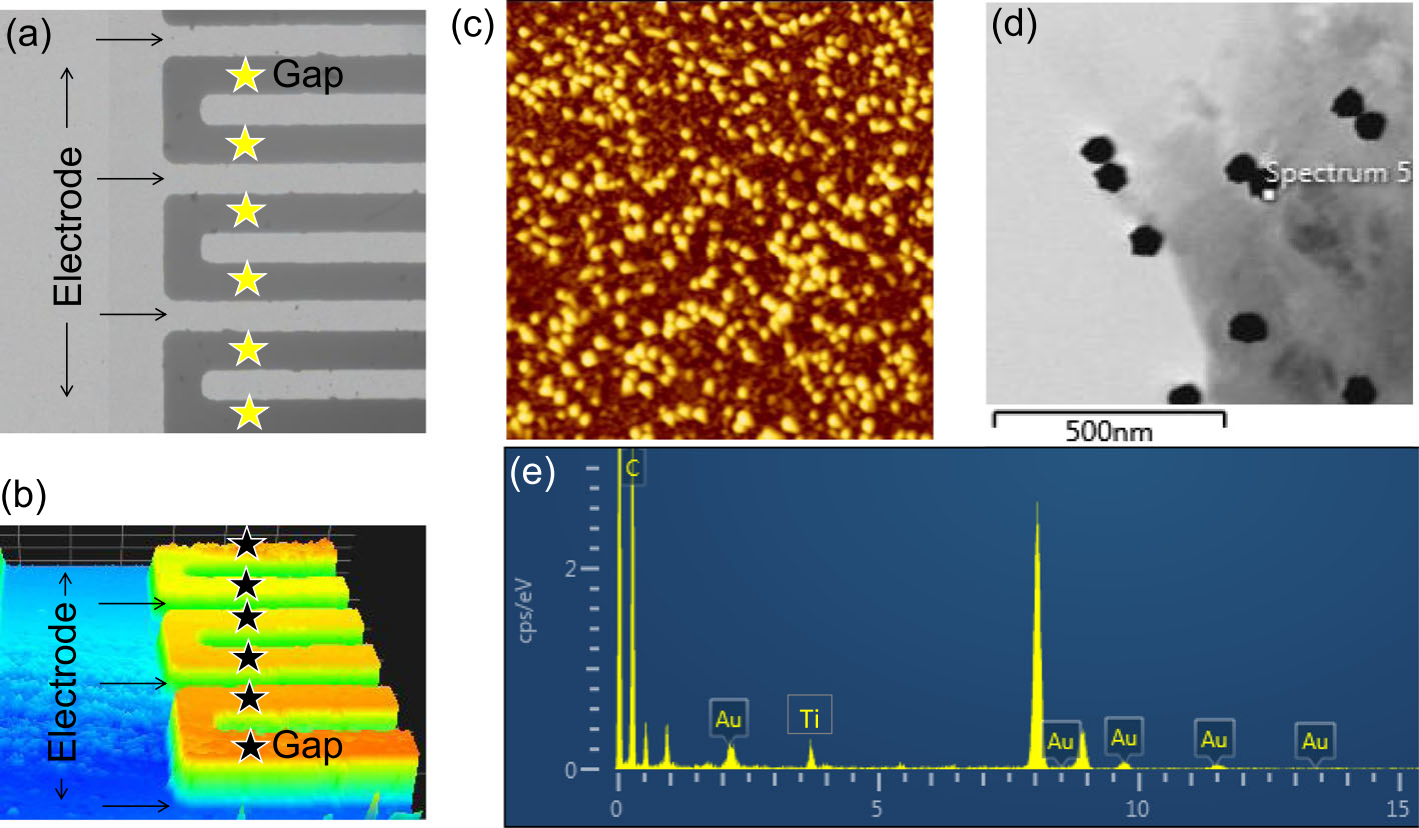
**a** High-power microscopy image on IDE surface. Images were captured at × 50. **b** 3D nanoprofiler image on IDE surface. Images were captured at × 50. Electrode and gap regions are shown. The gaps are indicated by stars. Uniform arrangements indicate the successful fabrication. **c** Atomic force microscopy image. AFM displays a clear discrimination between TiO_2_ and GNS by dark and bright spots, respectively. **d** Field-emission transmission electron microscopy image. **e** Energy dispersive X-ray analysis. Indicated the elements found on the surface

Figure 1 shows the schematic representation of detecting SCC-Ag on IDE-TiO_2_ sensing surface. As displayed in Fig. 1a, initially, IDE sensing surface was coated with TiO_2_ and then antibody was immobilized with or without GNS conjugation. These antibody-modified surfaces were used to detect the level of SCC-Ag. Before performing the detection, the conjugation of GNS with antibody was confirmed by UV-Vis spectroscopy. The GNS scanning profiles with the desired wavelength range before and after conjugation with antibody were determined. It was clearly seen that after immobilization, the shift was moved from 535 to 545 nM (Fig. 1b). This result confirms the conjugation of antibodies on the surface of the GNS. On the other hand, the fabricated sensing surface was observed morphologically. Figure 2a displays the high-power microscopy image, whereas Fig. 2b describes the image captured from 3D nanoprofiler imaging. Both imaging profiles are clearly shown with gap and electrode regions, which form the fingers. The arrangement of the gaps and fingers appeared to be uniform and intact.

### Comparison on Antibody and GNS Antibody Immobilization TiO_2_-IDE Sensing Surfaces

**Fig. 3:**
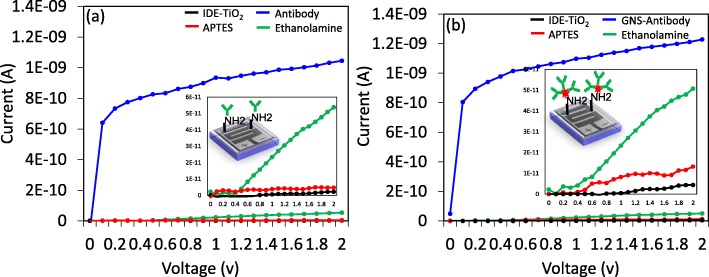
Immobilization processes on IDE-TiO_2_ surfaces. **a** With antibody. **b** With GNS antibody. Surface modifications were started by 3% APTES, followed by EDC and NHS activation to immobilize the antibody; 1 M of ethanolamine was used to block the unattached antibody region. A linear sweep voltage of 0 to 2 V at 0.01 V step voltage was followed for the measurements. Proper changes in current after each immobilization were confirmed the binding of antibody and GNS antibody on the sensing surfaces

SCC-Ag was detected on TiO_2_-IDE surface by antibody- or GNS antibody-immobilized surfaces. The attachment of GNS on TiO_2_ surface was confirmed by AFM, SEM observations and EDX analysis (Fig. 2c). Under AFM observation, a clear discrimination has been noticed between TiO_2_ and GNS by dark and bright spots, respectively. This was supported by SEM and EDX analyses, in which prominent gold and moderate titanium peaks were observed. These results evidence the occurrence of GNS on the TiO_2_ surface. Figure 3 shows the immobilization processes of antibody and GNS antibody on the amine-modified IDE-TiO_2_ sensing surfaces. TiO_2_-modified IDE sensing surface shows the current level as 4.65E−12 (Fig. 3a). After adding APTES, the current level was increased to 5.37E−11; this increment in current indicated that the surface was modified into amine by APTES. When the antibody was immobilized, the current level was changed from 5.375E−11 to 1.05E−9. The difference in the current was noticed as 1.04E−9 (Fig. 3a). This immobilization happened due to the chemical interaction of the amine group from the APTES and COOH group in the antibody [18]. The changes in the current confirmed the binding of antibody on the APTES modified surface. After that, the remaining surface was covered by 1 M ethanolamine to reduce the biofouling effect from the non-specific binding of biomolecules on the sensing surface. Similarly, GNS antibody was immobilized on TiO_2_-IDE surface, and when GNS antibody was immobilized on the APTES-modified surface, the current level was increased from 4.41E−12 to 1.23E−9 (Fig. 3b). It was clearly found that when the antibody was immobilized on the GNS surface, it shows the higher response on the amine-modified surface. This might be due to the larger number of antibodies bind on the surface of single GNS and the strong binding of this complex on the amine-modified surface. This binding happened due to the amino terminal group in the APTES displace the citrate groups on GNS and chemically fixed on the APTES-modified IDE surface [34]. It is well known that the detection of biomolecules on the sensing surfaces mainly depends on two factors, namely, binding affinity of interactive molecules and the proper surface immobilization of molecules on the sensing surface. Higher biomolecular immobilization on the sensing surface drastically improved the detection of a target at its lower level. In this research, GNS was used to immobilize anti-SCC-Ag antibody on IDE-TiO_2_ surface in order to enhance the chance of higher antibody binding, which leads the efficient SCC-Ag detection.

### Comparative Detection of SCC-Ag on IDE-TiO_2_ Surface by Antibody or GNS Antibody

**Fig. 4:**
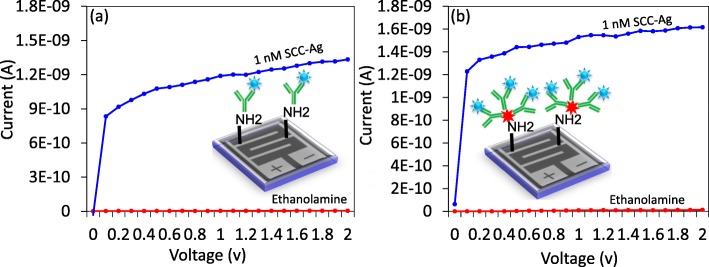
SCC-Ag detection with **a** antibody and **b** GNS antibody. Tested on IDE-TiO_2_ surfaces with the above steps until 1 M of ethanolamine blocking. A linear sweep voltage of 0 to 2 V at 0.01 V step voltage was followed for the measurements. After interacting 1 nM of SCC-Ag, the current levels were increased in both cases; at the same time, it shows a higher current change with GNS antibody

Since antibody GNS shows the efficient immobilization on IDE-TiO_2_ surface, the similar 1 nM concentration of SCC-Ag was detected on both antibody and GNS antibody surfaces and compared the changes in the current level. Figure 4a shows 1 nM of SCC-Ag detection on an antibody-modified surface. Before performing the detection, the antibody-modified surface was covered by the blocking agent ethanolamine to avoid non-specific binding of biomolecules. Ethanolamine shows the current change as 4.65E−12. After adding 1 nM of SCC-Ag, the current level was increased to 1.33E−09. These current changes clearly indicated the binding of SCC-Ag to its antibody. In the case of GNS antibody surface, the ethanolamine shows the current level as 1.33E−11; after adding 1 nM of SCC-Ag, it was increased to 1.62E−09 (Fig. 4b). The current changes with GNS antibody were higher compared with only antibody-modified surface for a similar concentration of SCC-Ag. This might be due to the higher number of antibodies bound in IDE-TiO_2_ surface through GNS.

### Limit of Detection of SCC-Ag on IDE-TiO_2_ Surface by Antibody or GNS Antibody

**Fig. 5:**
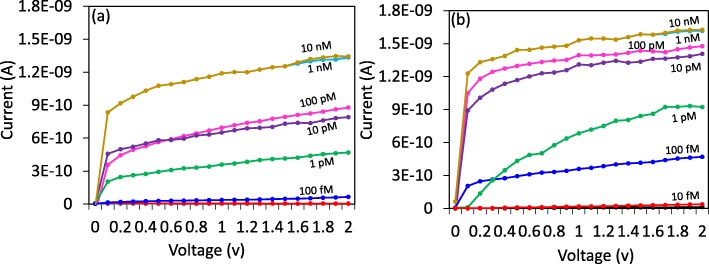
Dose-dependent interactions with **a** antibody and **b** GNS antibody on IDE-TiO_2_ surfaces. The surface is with the above steps until 1 M of ethanolamine blocking. A linear sweep voltage of 0 to 2 V at 0.01 V step voltage was followed for the measurements. SCC-Ag concentrations from 10 fM to 10 nM interacted on both surfaces, and the current changes were noticed. Washing was performed by five reaction volumes at each step using 10 mM PBS (pH 7.4). With an increase in SCC-Ag concentrations, the current levels were gradually increased in both cases. GNS antibody shows the current changes from 10 fM, while changes from 100 fM were noticed with only antibody. In both cases (antibody and GNS antibody), 1 nM SCC-Ag displayed the saturation. When the concentration is increased further, any significant changes in the current could not be observed

**Fig. 6:**
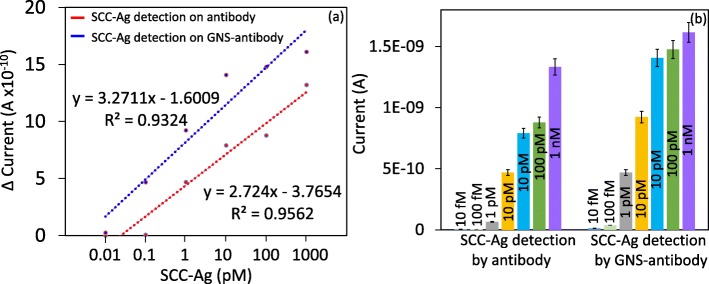
Comparison of current changes with different concentrations of SCC-Ag on antibody- and GNS antibody-modified surfaces. **a** Linear regression graph for the limit of detection of SCC-Ag. With antibody (red line) and with GNS antibody (blue line) are displayed. The limit of detection was found as 10 fM with GNS antibody and 100 fM with only antibody. **b** Current changes with SCC-Ag and antibody interaction. With all the concentrations, a higher level of current changes was found on GNS antibody surface. A linear sweep voltage of 0 to 2 V at 0.01 V step voltage was followed for the measurements. Error bar indicates the averaged values from triplicates (*n* = 3) with the standard deviations are in the range of ± 0.1 to 0.15 × 10^−9^ A. The limit of detection (LOD) was considered the lowest concentration of an analyte (from the calibration line at low concentrations) against the background signal (*S*/*N* = 3:1), in other words, LOD = standard deviation of the baseline + 3*σ*

Antibody- or GNS antibody-modified surfaces show a clear detection of SCC-Ag, and the limit of detection was estimated on both surfaces for comparison (Figure 5a, b). For that, the concentrations from 10 fM to 1 nM of SCC-Ag were diluted and dropped on these surfaces individually and noted the changes in the current. Figure 5a shows the different concentrations of SCC-Ag binding on the antibody-modified surface. After ethanolamine, 10 fM of SCC-Ag interacted, and there was no current change noticed. When increased the concentration to 100 fM, there was a minor change in the current from 4.65E−12 to 6.54E−11. Further, the concentrations were increased to 1 pM, 10 pM, 100 pM and 1 nM, and the current levels were increased as 4.69E−10, 7.91E−10, 8.78E−10 and 1.33E-09, respectively. These results are clearly indicating that with an increase in the concentrations, the binding is also increasing. The limit of detection was calculated based on 3*σ*, and it was at 100 fM (Fig. 6a).

The same concentrations of SCC-Ag interacted independently on GNS antibody-modified surfaces. When 10 fM of SCC-Ag was dropped on the surface, it clearly changed the current from 1.33E−11 to 3.74E−11. This result shows that even 10 fM of SCC-Ag can clearly interact with GNS antibody-immobilized surface, which cannot be detected in the case with only antibody. Moreover, when the concentrations were increased to 100 fM, 1 pM, 10 pM, 100 pM and 1 nM, the current levels were increased further to 4.69E−10, 9.23E−10, 1.41E−09, 1.48E−09 and 1.62E−09, respectively (Fig. 5b). The statistical calculations with the standard deviations are in the range of ± 0.1 to 0.15 × 10^−9^ A. When compared with the sensing on the above two surfaces, GNS antibody-modified surface shows the higher changes in the current with all the concentrations of SCC-Ag tested (Fig. 6b). Based on 3*σ*, it could find the limit of detection as 10 fM (Fig. 6a), this is 10 times better (lower) detection compared with only the antibody-modified surface. The statistical calculation with the standard deviations are in the range of ± 0.1 to 0.15 × 10^−9^ A. Previously, SCC-Ag has been evaluated on different nanomaterials, such as strontium nanoparticle and graphene; however, these surfaces displayed ~ 1000-folds lesser sensitivity compared with the current study [35].

### Selective Detection of SCC-Ag on Antibody/GNS Antibody-Modified Surfaces

**Fig. 7:**
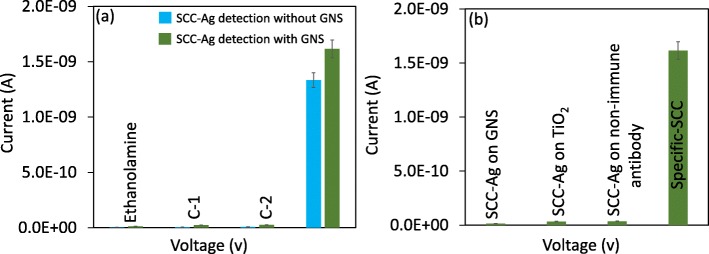
**a** Selective detection of SCC-Ag on antibody- and GNS antibody-modified surfaces. Interactions with C1-serpin and C-2-albumin were performed. The surface is with the above steps until 1 M of ethanolamine blocking. Values were averaged by triplicates. In both cases, antibody only recognised the SCC-Ag, indicating the specific detection. **b** Control measurements. Specificity interactions are compared with non-specific interactions. There were clear discriminations noticed. A linear sweep voltage of 0 to 2 V at 0.01 V step voltage was followed for the measurements. Error bar indicates the averaged values from triplicates (*n* = 3) with the standard deviations in the range of ± 0.1 to 0.15 × 10^−9^ A

Selective detection of SCC-Ag was compared with two control proteins, namely, serpin and albumin which are abundant in the bloodstream. Serpin is a protease inhibitor performing different human physiological functions and biological processes, whereas albumin accounts for 45 mg mL^−1^ and contributes 50–70% in the blood serum. As shown in the figure, 1 nM concentration of these two control proteins and SCC-Ag was dropped individually on the surfaces antibody or GNS antibody (Fig. 7a); it was clearly seen that the current changes were only noticed with SCC-Ag in both cases, indicating that antibody is able to recognize only SCC-Ag. There are no significant changes noticed in the current with the interaction of control proteins. This experiment confirms that the current experimental set-up can specifically detect/diagnose SCC-Ag. Further supports were rendered by other control experiments by the interactions of SCC-Ag with GNS alone and SCC-Ag with TiO_2_-IDE surface coated with non-immune antibody-labelled GNS. There were no significant changes in the current noticed compared with the specific interaction (Fig. 7b).

## Conclusion

Head and neck cancer is a common cancer that affects the areas of the mouth, throat and salivary glands. Diagnosing head and neck cancer with a suitable biomarker is mandatory to give the necessary treatment to the patients and improve their lifestyle. SCC-Ag has been found to be one of the important biomarkers for cancers; herein, SCC-Ag was detected on the titanium oxide-coated interdigitated electrode sensing surface (IDE-TiO_2_). Antibody for SCC-Ag was immobilized on IDE-TiO_2_ surface and detected the SCC-Ag. The detection limit was found as 100 fM, and further increment in the limit of detection was attained by conjugating the antibody with gold nanostar (GNS antibody). The limit of detection was improved by 10-folds (to 10 fM), this might be due to the larger number of antibody bound on the amine-modified TiO_2_ surface through GNS. Moreover, control experiments were carried out with two different proteins and not able to recognize by the anti-SCC-Ag, indicating the selective detection of SCC-Ag. The demonstrated IDE-TiO_2_ sensing surface helps to diagnose the head and neck cancer, a strategy can be followed for the earlier detection.

## Data Availability

All of the data are fully available without restriction.

## Abbreviations

16-MDA: 16-Mercaptoundecanoic acid
APTES: (3-Aminopropyl)triethoxysilane
GNS: Gold nanostar
IDE: Interdigitated electrode
PBS: Phosphate-buffered saline
RT: Room temperature
SCC-Ag: Squamous cell carcinoma antigen
SiO_2_: Silicon dioxide
TiO_2_: Titanium oxide
UV: Ultraviolet
